# Effect of Electro-Acupuncture and Moxibustion on Brain Connectivity in Patients with Crohn’s Disease: A Resting-State fMRI Study

**DOI:** 10.3389/fnhum.2017.00559

**Published:** 2017-11-17

**Authors:** Chunhui Bao, Di Wang, Peng Liu, Yin Shi, Xiaoming Jin, Luyi Wu, Xiaoqing Zeng, Jianye Zhang, Huirong Liu, Huangan Wu

**Affiliations:** ^1^Key Laboratory of Acupuncture and Immunological Effects, Shanghai University of Traditional Chinese Medicine, Shanghai, China; ^2^Life Sciences Research Center, School of Life Sciences and Technology, Xidian University, Xi’an, China; ^3^Outpatient Department, Shanghai Research Institute of Acupuncture and Meridian, Shanghai University of Traditional Chinese Medicine, Shanghai, China; ^4^Stark Neurosciences Research Institute, Indiana University School of Medicine, Indianapolis, IN, United States; ^5^Department of Gastroenterology, Zhongshan Hospital, Fudan University, Shanghai, China; ^6^Department of Radiology, Shanghai Mental Health Center, Shanghai Jiaotong University School of Medicine, Shanghai, China

**Keywords:** acupuncture, moxibustion, Crohn’s disease, fMRI, functional connectivity

## Abstract

Acupuncture and moxibustion have been shown to be effective in treating Crohn’s disease (CD), but their therapeutic mechanisms remain unclear. Here we compared brain responses to either electro-acupuncture or moxibustion treatment in CD patients experiencing remission. A total of 65 patients were randomly divided into an electro-acupuncture group (*n* = 32) or a moxibustion group (*n* = 33), and treated for 12 weeks. Eighteen patients in the electro-acupuncture group and 20 patients in the moxibustion group underwent resting-state functional magnetic resonance imaging at baseline and after treatment. Seed-based analysis was used to compare the resting-state functional connectivity (rsFC) between bilateral hippocampus and other brain regions before and after the treatments, as well as between the two groups. The CD activity index (CDAI) and inflammatory bowel disease questionnaire (IBDQ) were used to evaluate disease severity and patient quality of life. Electro-acupuncture and moxibustion both significantly reduced CDAI values and increased IBDQ scores. In the electro-acupuncture group, the rsFC values between bilateral hippocampus and anterior middle cingulate cortex (MCC) and insula were significantly increased, and the changes were negatively correlated with the CDAI scores. In the moxibustion group, the rsFC values between bilateral hippocampus and precuneus as well as inferior parietal lobe (IPC) were significantly elevated, and the changes were negatively correlated with the CDAI scores. We conclude that the therapeutic effects of electro-acupuncture and moxibustion on CD may involve the differently modulating brain homeostatic afferent processing network and default mode network (DMN), respectively.

## Introduction

Crohn’s disease (CD) is a chronic inflammatory disease that most commonly affects the terminal ileum and neighboring colon, but can affect the entire digestive tract. The main clinical manifestations include abdominal pain, diarrhea and weight loss. Current therapeutic strategies are aimed at inducing and maintaining disease remission, preventing the incidence of complications, and preventing disease progression (Torres et al., 2016). Although medications such as mesalazine, glucocorticoids, and immunosuppressants are efficient in controlling the acute activity of disease, the side effects caused by long-term administration of these medications hinder their continuous usage (Clark et al., 2007; Saibeni et al., 2014). Emerging biological agents, for example tumor necrosis factor alpha (TNF-α) inhibitors, bring hope to some patients. However, the use of these agents places a heavy financial burden on them (Clark et al., 2007).

Recently, the importance of brain-gut axis dysfunction in the development and progression of inflammatory bowel disease (IBD) has drawn more attention (Bonaz and Bernstein, 2013; Al Omran and Aziz, 2014). Numerous recent studies have shown that structural and functional abnormalities of the brain may play crucial roles in the development of CD (Bao et al., 2015; Bao C. et al., 2016; Bao C. H. et al., 2016; Rubio et al., 2016; Thomann et al., 2016). As a principal structure of the limbic system, hippocampus affects the intestinal tract through multiple pathways, including the hypothalamic-pituitary-adrenal (HPA) axis, the vagus nerve and the immune system (Lathe, 2001). Studies using animal models of chemically-induced CD have revealed changes in the excitability and behavior of the central nervous system, such as activation of hippocampal microglia, changes in glutamate transmission and neuronal plasticity, increased levels of TNF-α, inducible nitric oxide synthase (iNOS), and nitrites in the hippocampus (Riazi et al., 2008; Heydarpour et al., 2016), and declined neuronal regeneration (Riazi et al., 2015; Zonis et al., 2015). These findings provide evidence for potential interaction between the hippocampus and intestinal inflammation as well as immunity. Previous neuroimaging studies have demonstrated alterations in gray matter volume and functional activity of the hippocampus in CD patients when compared to healthy volunteers (Bao et al., 2015; Bao C. et al., 2016; Bao C. H. et al., 2016). Hence, the hippocampus may play a pivotal role in the development, progression and remission of CD.

As an important complementary therapy and alternative to standard medicine, acupuncture and moxibustion have been used for thousands of years in the prevention and treatment of different diseases, and have been widely used for gastrointestinal diseases globally (Schneider et al., 2007; Cheifetz et al., 2017). Randomized controlled trials have indicated that acupuncture and moxibustion can help control intestinal inflammation and improve CD patient quality of life (Joos et al., 2004; Bao et al., 2014), but the underlying mechanisms have not yet been fully elucidated. Previous evidence has suggested that the clinical outcome of acupuncture treatment relies on its modulation of central nervous system activity (Han et al., 1982; Takahashi, 2011; Huang et al., 2014). Electro-acupuncture is a form of acupuncture with improved therapeutic efficacy, in which small electrical currents at certain frequencies are passed through acupuncture needles. Moxibustion is a therapy that consists of burning dried mugwort on acupoints to generate warm stimulation on the local regions. Herb-partitioned moxibustion is a form of moxibustion in which herbs are ground into powder, prepared as a certain size of herbal disc using a mold, and the moxa cone is burned to warm the herb-partition, which stimulates acupoints. Electro-acupuncture and moxibustion are two stimulation approaches, generally producing similar clinical outcomes. However, in our previous study, we found that electro-acupuncture and moxibustion induced different brain responses in CD patients (Bao C. et al., 2016). The use of electro-acupuncture or moxibustion treatment can correct abnormal brain function in CD patients, and these changes are closely associated with clinical outcomes (Bao C. et al., 2016). Considering the principle role of the hippocampus in the brain-gut axis, it is important to investigate if electro-acupuncture and moxibustion treatment can affect the resting-state functional connectivity (rsFC) of the hippocampus in CD patients, and the potential differences between the two approaches.

Seed-based rsFC analysis can clarify the correlation of time series signals of low frequency oscillation between brain areas of interest (seeds) and other brain areas within the same neuroanatomical system. It has been used to evaluate the synchronization of neuronal activity in varied brain regions to study brain function, and identify functional networks associated with a seed region (Biswal et al., 1995; Fox and Raichle, 2007; Bullmore and Sporns, 2009). This method has also been used to investigate the mechanisms underlying several diseases and acupuncture and moxibustion treatment (Chen et al., 2015; Deng et al., 2016; Wang et al., 2016; Ning et al., 2017). This study aimed to investigate the effects of electro-acupuncture or moxibustion treatment on rsFC using bilateral hippocampus as seeds, to compare the differences in brain response between the two approaches, and to further explore the correlation between these changes and clinical outcomes in CD patients experiencing remission.

## Materials and Methods

### Subjects

This study was approved by the Ethical Committee of Yueyang Hospital of Integrated Translational Chinese and Western Medicine, Shanghai University of Traditional Chinese Medicine. It was registered in the clinical trials database (NCT01696838)^1^. All participants were informed and signed a consent form and the study was carried out in accordance with the Declaration of Helsinki (Edinburgh version, 2000).

A total of 65 CD patients from the Specialist Outpatient Department of IBD, Shanghai Research Institute of Acupuncture and Meridian, and the Endoscopy Center of Zhongshan Hospital, Fudan University, were recruited in this study. All patients received systemic and gastrointestinal screening, including colonoscopy and biopsy, by an experienced gastroenterologist from the Department of Gastroenterology, Zhongshan Hospital, Fudan University. Disease severity was scored according to the CD Index of Severity (CDEIS; Mary and Modigliani, 1989). In addition, data were collected for patient serum C-reactive protein level, erythrocyte sedimentation rate, and platelet count. Patient mental health was evaluated by a psychiatrist from Shanghai Mental Health Center according to DSM-IV criteria, and participants with psychological and psychiatric disorders were excluded.

Patients who met the following criteria were included for this study: 18–70 years of age; education history ≥6 years; right-hand dominant; disease remission >1 year; Crohn’s disease activity index (CDAI) score ≤150; CDEIS score <3; and no history of acupuncture or moxibustion treatment. Patients were excluded if they met any of the following criteria: serum C-reactive protein >10 mg/L; erythrocyte sedimentation rate >20 mm/h; platelet level >300 × 10^9^/L; presence of abdominal fistula or sinus tract; history of medical therapy with glucocorticoids, immunosuppressants, anti-TNF-α agents or other biologics, mental health medications, or opioids in the past 3 months; in a pregnant state; currently experiencing or had a history of mental/neurological disease, brain trauma, or loss of consciousness; with chronic damage or disease of the heart, liver, or kidneys; or had experienced tuberculosis, acute suppurative disease, or an infectious disease.

Patients under the following conditions were excluded from MRI examination: history of abdominal surgery related to CD; claustrophobia; presence of metal inside the body; or over 50 years of age (brain structure and function in these subjects have greater variation; Liu et al., 2016; Wu et al., 2016).

Participants taking mesalazine continued the drug administration with unchanged dosing during the study.

### Randomization

In this study, simple randomization was used. A random number table was generated using SPSS 16.0 statistical software by a non-related individual, and a random distribution table was passed to the experimental researchers. Participants that met the inclusion criteria were assigned to a random number sequentially according to their admission number. Subjects with odd random numbers were assigned to the electro-acupuncture group (*n* = 32) and those with even random numbers were assigned to the moxibustion group (*n* = 33). During treatment, patients in the two groups were admitted to separate treatment units in the study center to avoid interpersonal communication.

### Electro-Acupuncture and Moxibustion Treatments

**Figure 1:**
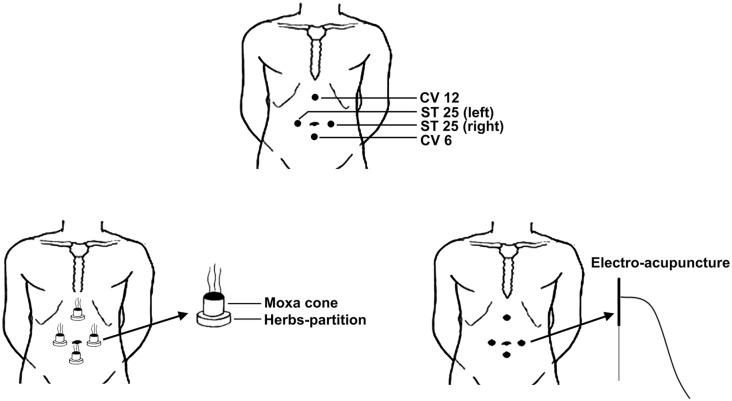
Locations of selected acupoints and schematic diagram of electro-acupuncture and moxibustion treatment. The acupoints were ST25 (*stomach, Tianshu, bilateral*), CV6 (*conception vessel, Qihai*) and CV12 (*Zhongwan*).

According to our previous studies (Bao et al., 2014; Bao C. et al., 2016), four acupoints were selected for both groups, including bilateral ST25 (*stomach, Tianshu, bilateral*), CV6 (*conception vessel, Qihai*) and CV12 (*Zhongwan*; Figure 1).

#### Electro-Acupuncture Procedures

Following local disinfection, an acupuncture needle (0.30 × 40 mm; Hwato, Suzhou Medical Appliance Factory) was rapidly inserted through the skin, then slowly inserted an additional 20–25 mm into the subcutaneous region. After achieving a Qi sensation of acupuncture, the needles were connected to a Han’s Acupoint Nerve Stimulator (HANS-100). Two electrodes of the stimulators (not divided into positive and negative charge) were connected to the left ST25 and CV6, as well as the right ST25 and CV12. The two clips did not touch each other. Dense disperse waves (frequency 2/100 Hz, current intensity 1–2 mA) were used, and the needles were left in place for 30 min. Patients were given electro-acupuncture treatment once every other day (3 days per week) for a total of 12 weeks (36 total sessions).

#### Moxibustion Procedures

An herbal disc used in herb-partitioned moxibustion was composed of Monkshood, *Coptis chinensis, Radix aucklandiae*, *Carthamus tinctorius*, Salvia and *Angelica sinensis*. Each herbal disc contained 2.8 g raw power. The powder was mixed with maltose (3 g) and made into a paste with warm water. The herbal disc was prepared to a uniform size using a mold (23 mm in diameter, 5 mm in thickness). Refined pure mugwort (size, 18 mm × 200 mm; Hanyi; Nanyang Hanyi Moxibustion Technology Development Co., Ltd.) was cut to a 16 mm height (final weight 1.8 g) for herb-partitioned moxibustion treatment. For each acupoint, two moxa-cones were burnt. The therapeutic course was the same as electro-acupuncture treatment (36 sessions over 12 weeks).

### Outcome Measurement

CDAI (Best et al., 1979) is a globally accepted index for accurate evaluation of the disease severity and therapeutic outcome for CD patients. CDAI ≤150 was identified as remission, and CDAI >150 was identified as active disease. The inflammatory bowel disease questionnaire (IBDQ; Irvine et al., 1994) is a questionnaire used to efficiently evaluate the health-related quality of life in adults with IBD. It contains 32 questions, with each question having a seven-point Likert scale. The total score ranges from 32 to 224 points. A higher score represents a better quality of life. Patients were assessed at baseline and after the 12-week treatment.

### MRI Protocol and Image Acquisition

The MRI experiments were conducted using a 3.0 Tesla clinical scanner (MagnetomVerio, Siemens, Erlangen, Germany) in the Department of Radiology, Shanghai Mental Health Center, Shanghai Jiaotong University (Shanghai, China). The MRI scans were performed 3 days before the treatment and 3 days after the end of treatment. During the MRI scan, all participants were instructed to relax and keep their eyes closed but not to fall asleep and not to think of anything in particular. Resting-state fMRI was performed with an echo-planar imaging sequence (TR/TE: 2000 ms/30 ms, flip angle = 90°, field of view (FOV) = 240 mm × 240 mm, matrix size = 240 × 240, in-plane resolution = 1 mm × 1 mm, slice thickness = 5 mm with no gaps and 32 slices). High resolution three-dimensional T_1_-weighted imaging was performed with a multi-echo magnetization-prepared rapid gradient-echo (MPRAGE) sequence with the following parameters: TR/TE = 2300/2.98 ms, FOV = 256 mm × 256 mm, matrix size = 256 × 256, flip angle = 9°, slice thickness = 1.0 mm and 176 slices.

### Image Data Processing

The resting-state fMRI datasets were processed using statistical parametric mapping software (SPM8)^2^. The first 10 images of each dataset were removed due to instability of the initial MRI signal and adaptation of participants to the environment. The remaining images were analyzed. Briefly, images from each subject were slice-time corrected and head-motion corrected. After realignment, all images were normalized to the standard Montreal Neurological Institute (MNI) template, and then resampled into 3 × 3 × 3 mm^3^ resolution. Next, the images were smoothed with a Gaussian kernel of 6 mm full-width at half maximum (FWHM). Six head motion parameters, signals of the cerebrospinal fluid (CSF), and signals from white matter were used as nuisance covariates to reduce the effects of head motion and non-neuronal blood oxygenation level-dependent (BOLD) fluctuations (Graybiel, 2008; Montembeault et al., 2012). To reduce low-frequency drift and high-frequency respiratory and heart rhythms, the linear trend in the fMRI data was removed, and the images were temporally bandpass filtered (0.01–0.1 Hz).

### FC Analysis

**Figure 2:**
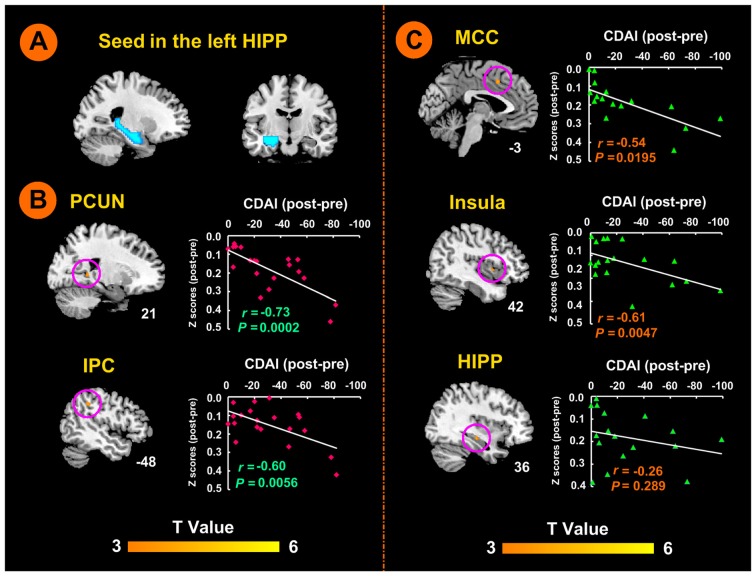
Left hippocampal seed locations and brain responses to electro-acupuncture and moxibustion treatment. **(A)** Seed region in the left hippocampus. **(B)** Brain regions showing an increase of rsFC with the left hippocampus following moxibustion compared to electro-acupuncture treatment, and the correlation between rsFC and CDAI scores. **(C)** Brain regions showing less of an increase of rsFC with the left hippocampus after electro-acupuncture compared to moxibustion treatment, and the correlation between rsFC and CDAI score changes. CDAI, Crohn’s disease activity index; HIPP, hippocampus; IPC, inferior parietal cortex; MCC, middle cingulate cortex; PCUN, precuneus; rsFC, resting-state functional connectivity.

Seed-based, whole-brain rsFC analyses were performed using the Conn Toolbox (Whitfield-Gabrieli and Nieto-Castanon, 2012). In this study, the WFU Pickatlas Tool^3^ was used to define the bilateral hippocampus, as demonstrated in previous studies (Rasetti et al., 2014; Duan et al., 2017; Figure 2A). Correlation maps were created by computing the correlation coefficients between the mean BOLD time course from the seed region and those from all other brain voxels. Correlation coefficients were then converted to *z* values using Fisher’s r-to-z transformation to improve the normality.

### Statistical Analysis

Twenty patients in each group were scheduled for MRI scans at baseline and at the end of treatment. In the electro-acupuncture group, one patient had head motion during the MRI scan, and one patient was not scanned due to a conflict of schedule. Therefore, a total of 18 MRI datasets from the electro-acupuncture group and 20 from the moxibustion group were included for final analysis. Among the 25 patients who were not scheduled for MRI scans, 12 patients were in the electro-acupuncture group (five were older than 50 years of age, three had metal implants in their body, and four had refused to receive the MRI scan) and 13 patients were in the moxibustion group (five were older than 50 years of age, six had metal implants in their body, and two had refused to receive the MRI scan).

A paired *t*-test was used to evaluate rsFC alterations induced by different treatments within the two groups (electro-acupuncture and moxibustion; *P* < 0.05, false discovery rate (FDR) corrected and the cluster size ≥48). The differences of rsFC between the two groups were evaluated using a two sample *t*-test (post-treatment minus pre-treatment; *P* < 0.05, FDR corrected and the cluster size ≥48). To analyze the relationships between changes in rsFC and disease severity (CDAI scores), Pearson’s correlation was then calculated in patient groups with *P* < 0.05 using the Bonferroni correction.

SPSS 16.0 software (SPSS Inc., Chicago, IL, USA) was used for statistical analysis of the clinical variables. All clinical data were subjected to an intention-to-treat (ITT) analysis using the last observation carried forward approach. Measurement data that were normally distributed were compared by independent sample *t*-test between groups. Measurement data without normal distribution were subjected to non-parametric tests. Enumeration data were compared between groups using a Chi-squared test or Fisher’s exact test. Two-tailed tests were applied for all analyses and *P* < 0.05 was considered statistically significant.

## Results

### Clinical Outcome

Table 1 shows a comparison of clinical and demographic variables of CD patients between the two groups who completed MRI scanning. There was no significant difference in the gender, age, height, weight, disease duration, concomitant medication (mesalazine), or the CDAI and IBDQ scores between the two groups of patients (all *P* > 0.05). Table 2 shows that the CDAI scores were significantly reduced (*P* < 0.01), while the IBDQ scores were significantly increased (*P* < 0.01) following treatment in both groups. There was no significant difference in the CDAI (*P* = 0.634) or IBDQ scores (*P* = 0.93) between the two groups after treatment.

**Table 1:** Demographic and clinical characteristics of CD patients at baseline in each group.

	Electro-acupuncture group (*n* =18)	Moxibustion group (*n* =20)	Statistical value	*P* value
Age (years), mean ± SD	30.89 ± 6.09	29.35 ± 7.73	*t* = −0.676	0.503
Gender (male/female), *n*	13/5	13/7	*X*^2^ = 5.158	0.023
Concomitant medication (mesalazine, yes/no), *n*	15/3	20/0	*X*^2^ = 26.947	0.000
Height (cm), mean ± SD	170.44 ± 6.53	170.00 ± 7.77	*t* = −0.190	0.851
Weight (kg), mean ± SD	58.56 ± 9.14	56.85 ± 8.53	*t* = −0.595	0.556
Disease duration (years)	5.42 ± 4.22	5.93 ± 3.80	*t* = 0.391	0.698
CDAI	78.28 ± 40.95	71.85 ± 40.84	*t* = −0.484	0.631
IBDQ	171.72 ± 20.17	176.10 ± 32.23	*t* = 0.495	0.623
HADS-A	6.33 ± 6.33	5.35 ± 3.62	*t* = −0.894	0.377
HADS-D	5.61 ± 3.16	3.65 ± 3.82	*t* = −1.713	0.095

*CD, Crohn’s disease; CDAI, Crohn’s disease activity index; HADS-A, Hospital Anxiety and Depression Scale-Anxiety; HADS-D, Hospital Anxiety and Depression Scale-Depression; IBDQ, inflammatory bowel disease questionnaire; SD, standard deviation*.

**Table 2:** Clinical outcome measurements.

Items	Electro-acupuncture group (*n* = 18)	Moxibustion group (*n* = 20)
**CDAI**
Baseline, mean ± SD	78.28 ± 40.95	71.85 ± 40.84
Post-treatment, mean ± SD	51.53 ± 31.80	40.90 ± 27.78
*T* value	6.876	6.584
*P* value	0.000	0.000
Changes from baseline to post-treatment	−26.74 ± 29.23	−30.95 ± 24.83
*T* value	−0.479	
*P* value	0.634	
**IBDQ**
Baseline, mean ± SD	171.72 ± 20.17	176.10 ± 32.23
Post-treatment, mean ± SD	187.18 ± 18.69	192.05 ± 21.45
*T* value	42.486	40.048
*P* value	0.000	0.000
Changes from baseline to post-treatment	15.44 ± 18.02	15.95 ± 17.28
*T* value	0.088	
*P* value	0.930	

*CDAI, Crohn’s disease activity index; IBDQ, inflammatory bowel disease questionnaire; SD, standard deviation, compared with baseline*.

Additionally, the clinical outcomes of either male or female population was similar to those when the both populations were combined (Supplementary Tables S1–S4).

### Modulation of rsFC between Left Hippocampus and Other Brain Regions by Electro-Acupuncture and Moxibustion Treatment

There was no significant difference in the rsFC baseline characteristics between the two groups (*P* > 0.05). After the 12-week treatment, the electro-acupuncture group showed significant increases in rsFC values between the left hippocampus seed region (shown in Figure 2A) and the left anterior middle cingulate cortex (MCC), the right insula, and the right hippocampus. In the moxibustion group, the rsFC values were significantly elevated between the left hippocampus seed region and the right precuneus (PCUN), and the left inferior parietal lobe (IPC; *P* < 0.05, FDR corrected). We did not detect any brain region that showed reduced rsFC with the left hippocampus (*P* < 0.05, FDR corrected).

As shown in Figure 2B, the moxibustion group showed a greater increase in the rsFC between the left hippocampus seed and the right PCUN and left IPC compared to the electro-acupuncture group (*P* < 0.05, FDR corrected). As shown in Figure 2C, the electro-acupuncture showed a greater increase in the rsFC between the left hippocampus seed and left MCC, right insula and right hippocampus compared to the moxibustion group (*P* < 0.05, FDR corrected).

In the moxibustion group, the increase of rsFC between the left hippocampus seed and the right PCUN and left IPC was negatively correlated with a reduction in the CDAI score (*r* = −0.73, *P* < 0.001; *r* = −0.60, *P* < 0.01; Figure 2B). In the electro-acupuncture group, the increase of rsFC between the left hippocampus seed and the left anterior MCC and right insula was negatively correlated to the reduction in the CDAI score (*r* = −0.54, *P* < 0.05; *r* = −0.61, *P* < 0.005; Figure 2C); the increase of rsFC between the left hippocampus seed and the right hippocampus was not significantly correlated to the reduction in the CDAI score (*r* = −0.26, *P* > 0.05; Figure 2C).

### Modulation of rsFC between Right Hippocampus and Other Brain Regions by Electro-Acupuncture and Moxibustion Treatment

**Figure 3:**
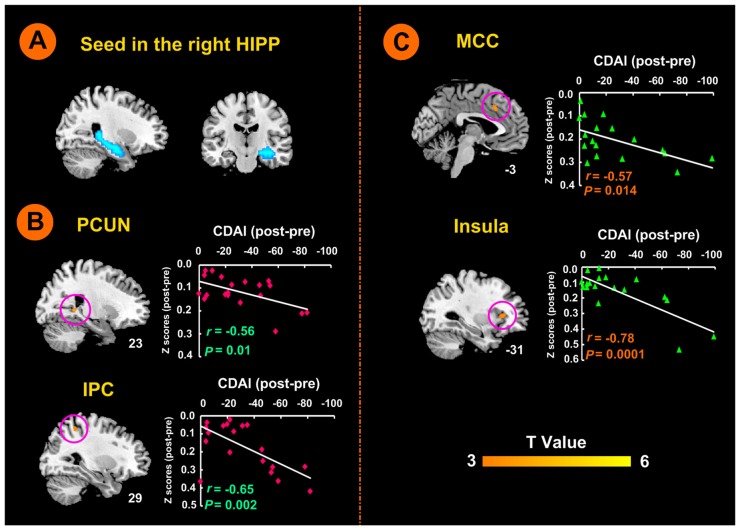
Right hippocampal seed locations and brain responses to electro-acupuncture and moxibustion treatment. **(A)** Seed region in the right hippocampus. **(B)** Brain regions showing an increase of rsFC with the right hippocampus following moxibustion compared to electro-acupuncture treatment, and the correlation between rsFC and CDAI scores. **(C)** Brain regions showing less of an increase of rsFC with the right hippocampus after electro-acupuncture compared to moxibustion treatment, and the correlation between rsFC and CDAI score changes. CDAI, Crohn’s disease activity index; HIPP, hippocampus; IPC, inferior parietal cortex; MCC, middle cingulate cortex; PCUN, precuneus; rsFC, resting-state functional connectivity.

There was no significant difference in the rsFC baseline characteristics between the two groups (*P* > 0.05). After the 12-week treatment, the rsFC value in the electro-acupuncture group was significantly increased between the right hippocampus seed region (shown in Figure 3A) and the left anterior MCC and the left insula. In the moxibustion group, the rsFC value was significantly elevated between the right hippocampus seed region and the right precuneus (PCUN), and the right IPC (*P* < 0.05, FDR corrected). We did not detect any brain region that showed reduced rsFC with the right hippocampus (*P* < 0.05, FDR corrected).

As shown in Figure 3B, the moxibustion group showed a greater increase in the rsFC between the right hippocampus seed and the right PCUN and right IPC compared to the electro-acupuncture group (*P* < 0.05, FDR corrected). As shown in Figure 3C, the electro-acupuncture showed a greater increase in the rsFC between the right hippocampus seed and left MCC and left insula compared to the moxibustion group (*P* < 0.05, FDR corrected).

In the moxibustion group, the increase of rsFC between the right hippocampus seed and the right PCUN and right IPC was negatively correlated with a reduction in the CDAI score (*r* = −0.56, *P* = 0.01; *r* = −0.65, *P* < 0.01; Figure 3B). In the electro-acupuncture group, the increase of rsFC between the right hippocampus seed and the left anterior MCC and left insula was negatively correlated to the reduction in the CDAI score (*r* = −0.57, *P* < 0.05; *r* = −0.78, *P* < 0.001; Figure 3C).

## Discussion

Our results demonstrate that electro-acupuncture and moxibustion treatments resulted in similar improvements in clinical outcomes but different changes in brain connectivity in CD patients in remission. Electro-acupuncture primarily modulates the homeostatic afferent processing network, whereas moxibustion treatment mainly regulates the default mode network (DMN). Our findings suggest that electro-acupuncture and moxibustion may help improve clinical outcomes through different central integration patterns, and provide new insight for understanding the therapeutic mechanisms of these two approaches.

Previous studies have showed abnormality in the homeostatic afferent processing network and DMN in CD patients in remission (Bao et al., 2015; Bao C. et al., 2016). The homeostatic afferent processing network participates in the regulation of visceral sensation, pain, emotion and homeostasis. Non-painful and painful stimulation from the viscera and body, as well as emotional stimulation, can activate this network (Mayer et al., 2006; Van Oudenhove et al., 2007). The DMN is mainly involved in monitoring external environment, which is a spontaneous cognitive process (Buckner et al., 2008; Mantini and Vanduffel, 2013).

Following electro-acupuncture treatment, the functional connectivity between the bilateral hippocampus and the homeostatic afferent processing network (insula and anterior MCC) was enhanced. In addition, the clinical outcomes of electro-acupuncture treatment were significantly correlated with increases in rsFC between the bilateral hippocampus and these two areas. The insula is considered the “interoceptive cortex” that processes different modalities of sensory information regarding the internal state of the body (Van Oudenhove et al., 2007). The MCC is a component of the medial pain system that processes pain-related emotional and cognitive information. The anterior MCC and subcortical structures, such as hypothalamus and PAG, constitute the descending pain modulation system, which functions in processing endogenous pain. The system mediates nociceptive information at the level of the spinal dorsal horn and controls the perception of pain (Wiech et al., 2008; Blankstein et al., 2010). Electro-acupuncture may help restore the homeostatic afferent processing network function, improve the cerebral integration of the gastrointestinal afferents, and balance homeostasis in CD patients by modulating the functional connectivity between brain regions related to visceral sensation, pain, and internal perception. This result is consistent with our previous findings (Bao C. et al., 2016) as well as results of a neuroimaging study that used electro-acupuncture for the treatment of functional dyspepsia (Zeng et al., 2012).

Following moxibustion treatment, the functional connectivities between the bilateral hippocampus and the DMN (PCUN and IPC) were enhanced, which were positively correlated with the clinical outcomes. The PCUN and IPC are two important components of the DMN. The PCUN plays a pivotal role in the processing of emotions associated with self-introspection and episodic memory (Maddock, 1999; Schneider et al., 2008), whereas the IPC is linked to concentration (Binder et al., 2009). During moxibustion treatment, the abdomen of the patient was always warm. The patient was pleased and felt positive regarding the warm stimulation generated by moxibustion treatment, which increased the attention of the patient on the abdomen. The warm stimulation is a key factor for the outcome of moxibustion treatment (Pach et al., 2009; Yi, 2009), and may mediate moxibustion-induced influences on DMN-associated brain regions. In summary, moxibustion treatment may enhance body attention, enhance functional connectivity between the bilateral hippocampus and the PCUN and IPC, and trigger the re-integration of the DMN, all of which may positively regulate gastrointestinal function in patients. These results are consistent with our previous findings (Bao C. et al., 2016).

The different effects of acupuncture and moxibustion on brain connectivity are likely due to a difference in their stimulation properties. Electro-acupuncture combines mechanical and electrical stimulation, while moxibustion is a kind of warm stimulation. Afferent input from electro-acupuncture stimulation ascends to thalamus and projects to multiple targets including the limbic system and cerebro-cerebellar (Hui et al., 2005), and were represented by the response of the homeostatic afferent processing network in CD patients. While the warm stimulation of moxibustion makes a subject to feel comfortable in the abdomen, resulting in a pleasure emotional experience, which may activate the DMN responsible for internal and external environmental monitoring and concentration. The input signals of both treatments may be carried primarily by mechanosensory, and pain and temperature sensation pathways. However, because acupuncture is shown to mainly activate Aβ and Aδ afferent fibers while the warm stimulus of moxibustion mainly activates C afferent fibers (Han, 2009, 2016), these two stimulus signals likely activate specific peripheral and central pathways and ultimately lead to the activation of different brain regions that underlie the observed differences in brain connectivity in CD patients.

The main limitation of this study is the lack of a positive control group (e.g., medication administration) or a negative control (e.g., placebo). Because of the progressive and recurring nature of CD, the Ethical Committee rejected our initial protocol to include a placebo control group, even if the subjects were all in remission. Therefore, we cannot rule out the non-specific effects of electro-acupuncture and moxibustion treatment. The ethical issue may be addressed in future through shortening treatment period and a positive control may also be considered in the future.

## Conclusion

Our study demonstrated that electro-acupuncture and moxibustion treatments were both effective in improving symptoms in CD patients in remission but they may have different brain mechanism by enhancing different brain connectivity. Electro-acupuncture treatment may modulate brain function mainly through the homeostatic afferent processing network (insula and anterior MCC), whereas moxibustion treatment may modulate the activity of the DMN (PCUN and IPC). These findings provide a new insight on the brain mechanism and clinical application of acupuncture and moxibustion.

## Author Contributions

CB, PL, HL and HW: study protocol and design; CB, YS, LW, JZ and XZ: data acquisition; PL, CB and DW: data analysis and interpretation; CB and DW: drafting of the manuscript; XJ and YS: manuscript revision.

## Conflict of Interest Statement

The authors declare that the research was conducted in the absence of any commercial or financial relationships that could be construed as a potential conflict of interest.

## Footnotes

^1^https://clinicaltrials.gov/

^2^http://www.fil.ion.ucl.ac.uk/spm

^3^http://www.nitrc.org/projects/wfu_pickatlas/

## Abbreviations

CD: Crohn’s disease
CDAI: Crohn’s disease activity index
DMN: default mode network
HADS: hospital anxiety and depression scale
HIPP: hippocampus
HCs: healthy control subjects
IBDQ: inflammatory bowel disease questionnaire
IPC: inferior parietal cortex
MCC: middle cingulate cortex
rsFC: resting-state functional connectivity.
