# Supplementary material for: Effect of Electro-Acupuncture and Moxibustion on Brain Connectivity in Patients with Crohn’s Disease: A Resting-State fMRI Study
Source: Front Hum Neurosci. 2017 Nov 17;11:559. doi: 10.3389/fnhum.2017.00559 (PMC5698267; doi:10.3389/fnhum.2017.00559)
Supplement: Supplementary file 1 [file Table_1.doc]

**Supplementary Table 1. Demographic and clinical characteristics of male patients with CD at baseline in each group.**

|  | Electro-acupuncture group (*n*=12) | Moxibustion group (*n*=13) | Statistical value | *P* value |
| --- | --- | --- | --- | --- |
| Age (years), mean ± SD | 31.75 ± 5.94 | 30.92 ±8.54 | *t* = -0.279 | 0.783 |
| Concomitant medication (mesalazine, yes/no), n | 10/2 | 11/2 | *X^2^* = 0.000 | 1.000 |
| Height (cm), mean ± SD | 173.58 ± 5.30 | 173.69 ± 5.15 | *t* = 0.052 | 0.959 |
| Weight (kg), mean ± SD | 61.33 ± 7.52 | 60.54 ± 8.13 | *t* = -0.253 | 0.802 |
| Disease duration (years) | 6.38 ± 4.00 | 7.15 ± 3.95 | *t* = 0.490 | 0.629 |
| CDAI | 78.53 ± 46.19 | 69.95 ± 40.34 | *t* = -0.496 | 0.625 |
| IBDQ | 168.67 ± 15.45 | 171.46 ±33.29 | *t* = *0.265* | 0.793 |
| HADS-A | 6.42 ± 21.15 | 5.85 ± 3.93 | *t* = -0.444 | 0.661 |
| HADS-D | 6.333 ± 2.84 | 3.69 ± 3.88 | *t* = -1.928 | 0.066 |

CD, Crohn’s disease; CDAI, Crohn’s disease activity index; HADS-A, Hospital Anxiety and Depression Scale-Anxiety; HADS-D, Hospital Anxiety and Depression Scale-Depression; IBDQ, inflammatory bowel disease questionnaire; SD, standard deviation.
