# Supplementary material for: Effect of Electro-Acupuncture and Moxibustion on Brain Connectivity in Patients with Crohn’s Disease: A Resting-State fMRI Study
Source: Front Hum Neurosci. 2017 Nov 17;11:559. doi: 10.3389/fnhum.2017.00559 (PMC5698267; doi:10.3389/fnhum.2017.00559)
Supplement: Supplementary file 2 [file Table_2.doc]

**Supplementary Table 2. Clinical outcome measurements of male patients with CD.**

| Items | Electro-acupuncture group (*n* = 12) | Moxibustion group (*n* = 13) |
| --- | --- | --- |
| CDAI | | |
| Baseline, mean ± SD | 78.53 ± 46.19 | 69.95 ± 40.34 |
| Post-treatment, mean ± SD | 47.67 ± 34.95 | 27.64 ± 7.67 |
| T value | 4.724 | 5.088 |
| *P* value | 0.001 | 0.000 |
| Changes from baseline to post-treatment | -30.87 ± 32.24 | -30.95 ± 27.23 |
| T value | -0.07 | |
| *P* value | 0.994 | |
| IBDQ | | |
| Baseline, mean ± SD | 168.67 ± 15.45 | 171.46 ±33.29 |
| Post-treatment, mean ± SD | 183.50 ± 15.34 | 188.23 ± 22.21 |
| T value | 41.434 | 30.555 |
| P value | 0.000 | 0.000 |
| Changes from baseline to post-treatment | 14.83 ± 17.80 | 16.77 ± 19.03 |
| T value | 0.262 | |
| *P* value | 0.796 | |

CD, Crohn’s disease; CDAI, Crohn’s disease activity index; IBDQ, inflammatory bowel disease questionnaire; SD, standard deviation, compared with baseline.
