# Supplementary material for: Effect of Electro-Acupuncture and Moxibustion on Brain Connectivity in Patients with Crohn’s Disease: A Resting-State fMRI Study
Source: Front Hum Neurosci. 2017 Nov 17;11:559. doi: 10.3389/fnhum.2017.00559 (PMC5698267; doi:10.3389/fnhum.2017.00559)
Supplement: Supplementary file 3 [file Table_3.doc]

**Supplementary Table 3. Demographic and clinical characteristics of female patients with CD at baseline in each group.**

|  | Electro-acupuncture group (*n*=6) | Moxibustion group (*n*=7) | Statistical value | *P* value |
| --- | --- | --- | --- | --- |
| Age (years), mean ± SD | 29.17 ± 6.55 | 26.43 ± 5.32 | *t* = -0.832 | 0.423 |
| Concomitant medication (mesalazine, yes/no), n | 5/1 | 7/0 | *X^2^* = 0.006 | 0.462 |
| Height (cm), mean ± SD | 164.17 ± 3.49 | 163.14 ± 7.34 | *t* = -0.312 | 0.761 |
| Weight (kg) | 53.00 ±10.20 | 50.00 ±3.74 | *t* = -0.727 | 0.482 |
| Disease duration (years) | 3.50 ± 4.32 | 3.64 ± 2.29 | *t* = 0.076 | 0.941 |
| CDAI | 77.77 ± 31.71 | 75.37 ± 44.77 | *t* = -0.109 | 0.915 |
| IBDQ | 177.83 ± 28.11 | 184.71 ± 30.67 | *t* = 0.419 | 0.683 |
| HADS-A | 6.17 ± 4.75 | 4.43 ± 2.99 | *t* = -0.803 | 0.439 |
| HADS-D | 4.17 ± 3.54 | 3.57 ± 3.99 | *t* = -0.282 | 0.783 |

CD, Crohn’s disease; CDAI, Crohn’s disease activity index; HADS-A, Hospital Anxiety and Depression Scale-Anxiety; HADS-D, Hospital Anxiety and Depression Scale-Depression; IBDQ, inflammatory bowel disease questionnaire; SD, standard deviation.
