# Supplementary material for: Effect of Electro-Acupuncture and Moxibustion on Brain Connectivity in Patients with Crohn’s Disease: A Resting-State fMRI Study
Source: Front Hum Neurosci. 2017 Nov 17;11:559. doi: 10.3389/fnhum.2017.00559 (PMC5698267; doi:10.3389/fnhum.2017.00559)
Supplement: Supplementary file 4 [file Table_4.doc]

**Supplementary Table 4. Clinical outcome measurements of female patients with CD.**

| Items | Electro-acupuncture group (*n* = 6) | Moxibustion group (*n* = 7) |
| --- | --- | --- |
| CDAI | | |
| Baseline, mean ± SD | 77.77 ± 31.71 | 75.37 ± 44.77 |
| Post-treatment, mean ± SD | 59.27 ± 25.34 | 44.43 ± 29.89 |
| T value | 5.730 | 3.932 |
| *P* value | 0.002 | 0.008 |
| Changes from baseline to post-treatment | -18.50 ± 22.28 | -30.94 ± 21.66 |
| T value | -1.019 | |
| *P* value | 0.330 | |
| IBDQ | | |
| Baseline, mean ± SD | 177.83 ± 28.11 | 184.71 ± 30.67 |
| Post-treatment, mean ± SD | 194.50 ± 23.94 | 199.14 ± 19.48 |
| T value | 19.901 | 27.047 |
| P value | 0.000 | 0.000 |
| Changes from baseline to post-treatment | 16.67 ± 20.09 | 14.43 ± 14.73 |
| T value | -0.232 | |
| *P* value | 0.821 | |

CD, Crohn’s disease; CDAI, Crohn’s disease activity index; IBDQ, inflammatory bowel disease questionnaire; SD, standard deviation, compared with baseline.
